# Work-related musculoskeletal symptoms among ear, nose and throat physicians in germany: a National survey

**DOI:** 10.1007/s00405-025-09663-8

**Published:** 2025-09-18

**Authors:** Antonia Lakomek, Theda Eichler, Tim Janßen, Moritz Meyer, Benedikt Höing, Kazim Shiraliyev, Stephan Lang, Börge Schmidt, Diana Arweiler-Harbeck

**Affiliations:** 1 Department of Otorhinolaryngology, Head and Neck Surgery, University Hospital Essen, Hufelandstraße 55, Essen, 45147 Germany; 2 Quality Management and Clinical Risk Management, University Hospital Essen, Hufelandstraße 55, Essen, 45147 Germany; 3 Department of Otorhinolaryngology, Facial, Head, Neck and Scull Base Surgery, Catholic Hospital Koblenz-Montabaur, Rudolf-Virchow-Str. 7-9, Koblenz, 56073 Germany; 4 Department of Medical Informatics, Biometry, and Epidemiology, University Hospital Essen, Hufelandstraße 55, Essen, 45147 Germany

## Abstract

**Background:**

Work-related musculoskeletal disorders (WRMD) are an increasing concern among surgeons, especially otorhinolaryngologists (ENT physicians), due to prolonged static postures and repetitive movements during clinical practice. However, data on the prevalence of WRMD among ENT physicians in Germany are lacking.

**Methods:**

A nationwide online survey was conducted among members of the German Society for ENT to assess demographic data, work-related factors, and musculoskeletal complaints. Data were analyzed using descriptive statistics.

**Results:**

A total of 751 ENT physicians participated (53% female; mean age 51 years). Lifetime prevalence of neck complaints was 93%, with 82% reporting symptoms in the past 12 months. Shoulders, upper and lower back were also frequently affected. More than a half (53%) reported limitations in professional activities, and 22% had taken sick leave due to these complaints. The prevalence of symptoms increased with years of professional experience. Female physicians reported higher rates of neck and upper back complaints compared to males.

**Conclusion:**

This study reveals a high prevalence and significant burden of WRMD among German ENT physicians, particularly in the neck and upper back regions. The findings emphasize the urgent need for preventive measures to improve occupational health and maintain professional longevity in this population.

## Introduction

Work-associated musculoskeletal complaints, internationally recognized as work-related musculoskeletal disorders (WRMD), are an increasing issue in surgery, affecting approximately 60–90% of surgeons [1, 2]. The burden of WRMD is reported not only by surgeons but also by physicians in general clinical practice [3].

In particular, the specialty of otolaryngology (ENT), where prolonged sitting at a microscope and twisted postures using an endoscope during surgery or in daily clinical practice are routine, can lead to postural misalignment. This shows a significant trend of WRMD affecting many practitioners [4].

Bolduc-Bégin et al., in a large-scale Canadian study through a survey of all ENT physicians, found that 97% of participants experienced musculoskeletal complaints at some point in their careers. Of these, 74% reported worsening symptoms during work, and 25% experienced a significant aggravation of symptoms after surgery. Furthermore, 15% of respondents stated that they had to take sick leave due to WRMD [5].

Similarly, Boyle et al. (2021) demonstrated the relevance of WRMD among ENT physicians in Ireland in a large-scale study, reporting a prevalence of 75.5% among respondents [6].

In Germany, this issue remains significantly underrepresented in the literature and in the daily life. To date, there are no studies investigating the prevalence of WRMD among ENT physicians in Germany.

It is important to determine the prevalence of complaints among ENT physicians to assess their relevance to daily practice. There are only a few individual studies that have addressed potential improvements for the daily routines of ENT physicians [7, 8]. These studies primarily focus on operative practice and do not provide a comprehensive overview of the broader issue within a large cohort.

The study presented here aims to assess the prevalence of WRMD among ENT physicians and surgeons in Germany. The goal is to raise awareness of this issue and to lay the groundwork for further studies and initiatives aimed at improving and preventing WRMD in Germany.

## Materials and methods

The study project was reviewed and approved by the local ethics committee (Ethics Committee of the University of Duisburg-Essen).For this survey, an online questionnaire was created using the program Evasys^®^ (version V9.0).

An online link to this questionnaire was sent to all ENT physicians who are members of the German Society for ENT (DGHNO) and are registered on the email distribution list of the newsletter. These doctors were asked to voluntarily complete the questionnaire anonymously, without any compensation.

After eight weeks, a friendly reminder email was sent, requesting the completion of the questionnaire. Following another eight weeks, the questionnaire was closed, and the analysis was started.

To reach as many participants as possible, the questionnaire was accompanied by a cover letter urging recipients to complete the survey even if they did not have any musculoskeletal complaints.

Completing the questionnaire took approximately five minutes for the participants.

### Development of the survey

The questionnaire was developed by an extensive review of the existing literature on occupational musculoskeletal disorders, associated risk factors in healthcare settings and research from other countries [3, 5, 6, 9]. A preliminary version of the questionnaire was administered to a small sample (*n* = 5) of healthcare professionals, representative of the target population. The pretest aimed to assess the feasibility of the questionnaire in terms of comprehensibility, length, and ease of use. Participants were asked to provide qualitative feedback on any ambiguities or difficulties encountered during completion. Based on this feedback, adjustments were made to optimize the clarity of certain questions and response options.

## Survey structure

The questionnaire utilized in this study was designed in cooperation with a specialist of the Institute of Medical Informatics, Biometry, and Epidemiology and a specialist of the assessment tool to systematically assess informed consent, demographic characteristics, work-related data, and musculoskeletal symptoms among healthcare professionals. It was structured into four distinct sections to ensure clarity and comprehensive data collection relevant to the study objectives.

The first section focused on obtaining informed consent from participants. Each participant was presented with a detailed information sheet outlining the purpose of the study, the voluntary nature of participation, and the anonymous processing of their data. Only participants who provided their consent were able to complete the questionnaire.

The second section captured demographic information. Participants were prompted to provide their age, height, weight, gender, weekly physical activity, and handedness.

The third section of the questionnaire addressed work-related factors. Participants were asked to report their total years of professional experience, average weekly working hours, average number of hours they spend in the operating room per week, professional specialization and workplace setting.

The final section focused on musculoskeletal symptoms, with a detailed assessment of their occurrence, characteristics, and impact. To ensure relevance to occupational factors, participants were asked to mark only those symptoms that could not be clearly linked to unrelated injuries, such as those caused by sports or similar activities. Participants were asked about symptoms in specific body regions, including the neck, shoulders, upper back, lower back, hips, knees, and feet/ankles. For each region, they were prompted to report symptoms within three distinct time frames: lifetime occurrence, symptoms within the past 12 months, and symptoms within the past 7 days. If symptoms were reported, participants were asked to further specify the nature of the complaints, such as muscle tension, restricted movement, or pain, as well as the laterality.

To assess the impact of these symptoms, additional itemsinquiring on limitations in daily activities, sports, or professional duties were implemented.

## Statistical analyses

Data were collected using the survey tool EvaSys^®^ (version V9.0) and exported in Excel spreadsheet format (Microsoft Excel 2016^®^). Descriptive statistics were used to analyse the data. Mean values were calculated and supplemented with standard deviation values, and distributions were presented both in percentages and absolute numbers.

## Results

### Demographic and work-related baseline data

A total of 751 participants took part in the study, of whom 53% (*n* = 395/750) were female and 47% (*n* = 355/750) male. The average age was 51 years. 90% (*n* = 676/750) were right-handed, 7% (*n* = 53/750) left-handed, and 3% (*n* = 21/750) were ambidextrous. On average, the participants had a BMI of 24. One third (35%, *n* = 262/751)) had a BMI of greater than or equal to 25, which is defined by the German Society for Obesity as overweight [10]. Regarding their workplace setting, 79% (*n* = 593/749) were employed in an outpatient medical practice, 5% (*n* = 40/749) worked in a medical care centre, and 15% (*n* = 116/749) were employed in a hospital. Of those working in hospitals, 8% (*n* = 61/749) were affiliated with a university hospital.

The majority of participants (55%) reported working across all listed subspecialties, with smaller groups focusing on nose/paranasal sinuses (18%) or ear (4%). The average work experience was 24 years, with nearly three-quarters (75%) having more than 15 years of experience. Participants worked an average of 40 h per week, with half (50%) reporting > 30–45 working hours and a quarter more than 45 (26%). Over half (54%) spending no time in the operating room, 35% were performing surgery until 10 h per week and 11% more than 10 h per week (Table 1).

**Table 1:** Demographic and work-related baseline data

Demographic data	
Gender (%) Female Male	52.60 (*n* = 395/750) 47.27 (*n* = 355/750)
Age (years) Mean (± SD)	51 (± 11)
Dominant hand Right Left Ambidextrous	90.13 (*n* = 676/750) 7.07 (*n* = 53/750) 2.8 (*n* = 21/750)
BMI Mean (± SD) <18.5 (%) 18.5–24.9 (%) >25 (%)	24.00 (± 5) 2.66 (*n* = 20/751) 62.45 (*n* = 469/751) 34.89 (*n* = 262/751)
**Work-related data**	
Workplace setting (%) Practice Medical care centre Hospital (not university) University hospital	79.17 (*n* = 593/749) 5.34 (*n* = 40/749) 7.34 (*n* = 55/749) 8.14 (*n* = 61/749)
Specialisation (%) No All of these Ear Nose/paranasal sinuses Soft tissue/tumour	55.20 (*n* = 433/751) 17.60 (*n* = 138/751) 3.83 (*n* = 30/751) 18.11 (*n* = 142/751) 5.23 (*n* = 41/751)
Work experience Mean (years), (± SD) <5 years (%) 5–15 (%) >15–30 (%) >30 (%)	23.87 (± 11) 3.99 (*n* = 30/751) 21.04 (*n* = 158/751) 46.87 (*n* = 352/751) 28.10 (*n* = 211/751)
Weekly working hours Mean (hours), (± SD) <15 h (%) 15–30 h (%) >30–45 h (%) >45 h (%)	40.33 (± 12) 1.46 (*n* = 11) 21.96 (*n* = 165) 50.33 (*n* = 378) 26.24 (*n* = 197)
Operating room time per week Mean (hours), (± SD) 0 h (%) <5 h (%) 5–10 h (%) >10–20 h (%) >20 h (%)	4.08 (± 7) 53.53 (*n* = 402/751) 17.04 (*n* = 128/751) 18.38 (*n* = 138/751) 7.99 (*n* = 60/751) 3.06 (*n* = 23/751)

The majority of participants (43%; *n* = 322/751) reported engaging in 2.5–5 h of physical activity per week, followed by 40% (*n* = 304/751) who exercised less than 2.5 h weekly. A smaller portion (12%; *n* = 88/751) reported more than 5 h of activity, while 5% (*n* = 37/751) indicated no physical activity at all.

### Work-related musculoskeletal symptoms and effects

A majority of ENT specialists reported musculoskeletal complaints in the neck, shoulders, and back. The neck was the most frequently affected anatomical region across all timeframes.

Overall, 93% of respondents indicated they had experienced neck-related complaints at some point in their careers, with 97% of these reporting tension, 81% pain, and 65% movement restriction. Within the last 12 months, 82% had neck issues, with similar distributions for tension (94%), pain (77%), and restriction (55%). In the last 7 days, 52% reported neck complaints, with tension (94%) and pain (68%) remaining the most common symptoms.

The lower back was the second most frequently affected region. Problems in these regions were prevalent, with 73% of respondents affected at some point, and 56% in the past year. Complaints included pain (91%) and tension (70%), with over half reporting restricted movement.

Shoulder complaints were also widespread, though less frequent than those affecting the neck. Overall, 67% reported shoulder issues at some point, with pain (85%) and tension (60%) being the dominant symptoms. Within the past year, 55% experienced shoulder complaints, and 32% reported them in the last 7 days.

Upper back issues were reported by 65% of participants over their careers, with a high proportion experiencing tension (89%) and pain (78%). These numbers declined slightly for the past 12 months (55%) and past week (32%), though tension and pain remained prominent.

In comparison, complaints related to the hips, knees, and feet/ankles were considerably less common (Table 2).

**Table 2:** Prevalence of symptoms in different anatomical regions

Anatomical regions/Type of complaints	Overall prevalence	12-month prevalence	7-days prevalence
**Neck** (%) Tension Pain Restriction	92.53 (*n* = 693/749) 96.67 (*n* = 670/693) 81.09 (*n* = 562/693) 65.37 (*n* = 453/693)	81.55 (*n* = 610/748) 94.10 (*n* = 574/610) 76.72 (*n* = 468/610) 55.25 (*n* = 337/610)	51.94 (*n* = 387/745) 93.59 (*n* = 362/387) 68.48 (*n* = 265/387) 42.38 (*n* = 164/387)
**Shoulder** (%) Tension Pain Restriction	67.28 (*n* = 502/746) 59.56 (*n* = 299/502) 84.86 (*n* = 426/502) 60.56 (*n* = 304/502)	54.48 (*n* = 407/747) 69.29 (*n* = 282/407) 84.77 (*n* = 345/407) 54.56 (*n* = 222/407)	31.57 (*n* = 234/741) 71.79 (*n* = 168/234) 77.78 (*n* = 182/234) 50.00 (*n* = 117/234)
**Upper back** (%) Tension Pain Restriction	65.04 (*n* = 482/741) 89.00 (*n* = 429/482) 78.01 (*n* = 376/482) 48.76 (*n* = 235/482)	55.13 (*n* = 409/742) 91.44 (*n* = 374/409) 76.99 (*n* = 315/409) 41.80 (*n* = 171/409)	31.52 (*n* = 232/736) 90.52 (*n* = 210/232) 68.10 (*n* = 158/232) 39.66 (*n* = 92/232)
**Lower back** (%) Tension Pain Restriction	72.79 (*n* = 540/742) 70.37 (*n* = 380/540) 90.93 (*n* = 491/540) 57.04 (*n* = 308/540)	55.89 (*n* = 413/739) 73.61 (*n* = 304/413) 87.16 (*n* = 360/413) 52.06 (*n* = 215/413)	31.14 (*n* = 231/742) 71.86 (*n* = 166/231) 85.71 (*n* = 198/231) 48.48 (*n* = 112/231)
**Hip** (%) Tension Pain Restriction	26.26 (*n* = 194/739) 36.60 (*n* = 71/194) 90.21 (*n* = 175/194) 53.61 (*n* = 104/194)	20.67 (*n* = 154/745) 46.10 (*n* = 71/154) 88.31 (*n* = 136/154) 57.79 (*n* = 89/154)	11.32 (*n* = 84/742) 44.05 (*n* = 37/84) 80.95 (*n* = 68/84) 59.52 (*n* = 50/84)
**Knee** (%) Tension Pain Restriction	33.02 (*n* = 245/742) 10.61 (*n* = 26/245) 94.29 (*n* = 231/245) 41.63 (*n* = 102/245)	22.39 (*n* = 167/746) 13.17 (*n* = 22/167) 96.41 (*n* = 161/167) 37.72 (*n* = 63/167)	11.14 (*n* = 82/736) 9.76 (*n* = 8/82) 93.90 (*n* = 77/82) 43.90 (*n* = 36/82)
**Foot/ankle** (%) Tension Pain Restriction	22.36 (*n* = 165/738) 21.21 (*n* = 35/165) 93.94 (*n* = 155/165) 43.03 (*n* = 71/165)	17.01 (*n* = 126/741) 23.81 (*n* = 30/126) 94.44 (*n* = 119/126) 42.86 (*n* = 54/126)	8.56 (*n* = 63/736) 22.22 (*n* = 14/63) 92.06 (*n* = 58/63) 39.68 (*n* = 25/63)

56% (*n* = 419/743) of the respondents reported being prevented at least once in their daily activities due to the specified complaints. Due to the complaints, 66% (*n* = 489/746) of the participants were already unable to take part in their regular sports activities. More than half (53%; *n* = 398/749) of the respondents reported that the complaints had affected their ability to perform work-related tasks. 22% (*n* = 166/744) of the participants have already had to take sick leave from work due to the complaints, with a concentration in the work experience group of 15–30 years of working (25%; *n* = 87/347). 11% (*n* = 84/748) of the respondents reported currently using or having used analgesics for the treatment of their complaints. 41% (*n* = 305/748) have undergone or are currently undergoing physiotherapeutic treatment, and 3% (*n* = 22/748) have already undergone surgery due to their complaints.

## Potential correlation between demographic and occupational baseline data and musculoskeletal complaints

There was no substantial difference in the prevalence of neck complaints between the group of ENT doctors who perform surgeries and those who do not (neck complaints no surgeons: 94% (*n* = 377/401) vs. surgeons 91% (*n* = 316/348). In the second most commonly affected region, the lower back, there was also no substantial difference between the group of operating and non-operating ENT doctors (lower back complaints no surgeons: 74%; *n* = 295/397 vs. surgeons 71%; *n* = 245/345).

Differences in the prevalence of muscular complaints were observed across the different work experience groups. While neck complaints were consistently high in all groups with the lowest prevalence in the group with less than five years of work experience (ranging from approximately 79% (< 5 years) to over 95% (> 15–30)), other anatomical regions showed more distinct variation.

Upper back complaints were less prevalent in the group with less than 5 years of experience (46%, *n* = 13/28), compared to those with more than 30 years of experience, where prevalence increased to approximately 66% (*n* = 137/209). Lower back complaints showed a steady increase with growing work experience. Starting at 45% (*n* = 13/29) in the less than five years group, prevalence rose steadily across all categories, reaching its highest value of 80% (*n* = 166/207) in those with more than 30 years of experience.

**Fig. 1:**
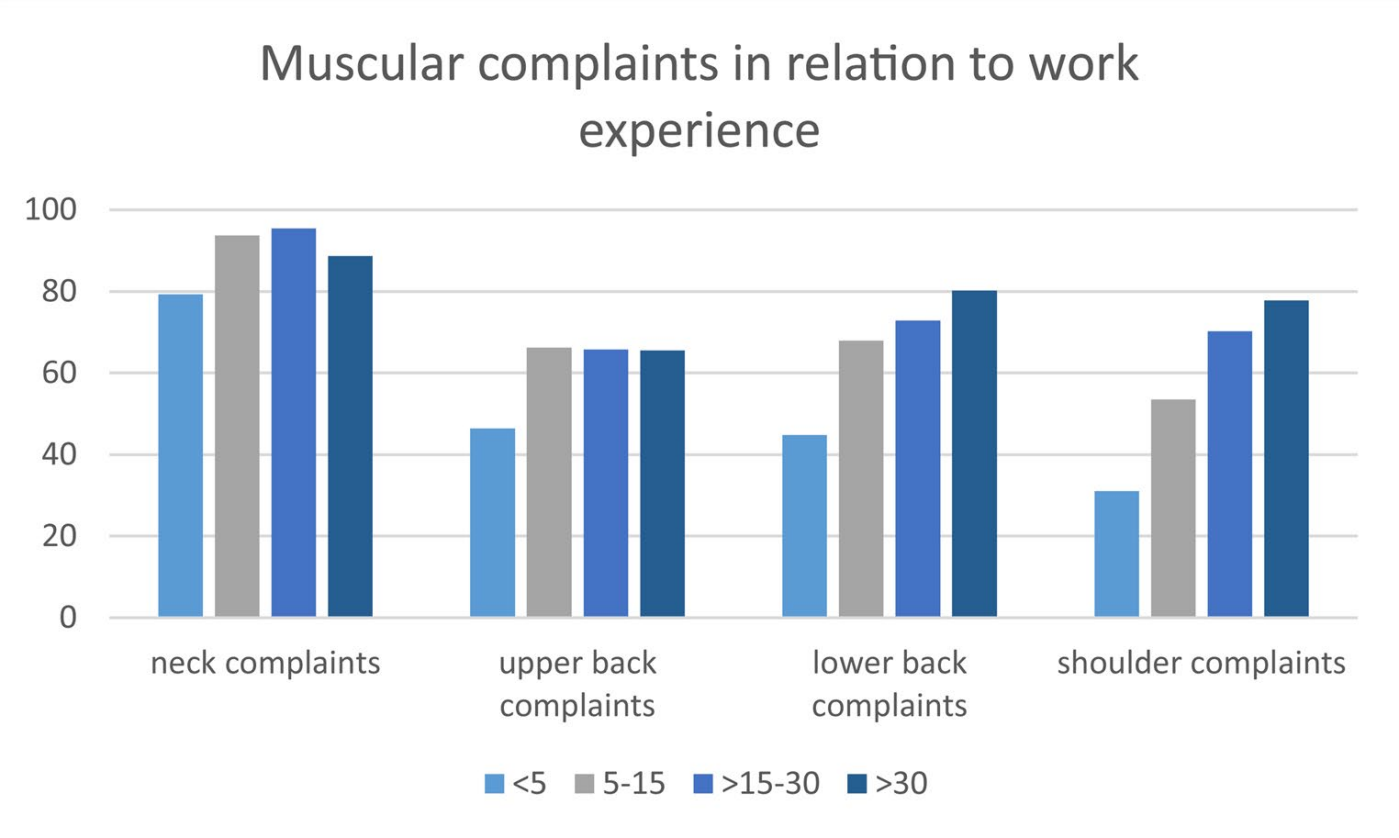
Muscular complaints in percent per group in relation to work experience in years

The highest difference between experience groups was observed in shoulder complaints (< 5 years 31%; *n* = 9/29 vs. >30 years 78%; *n* = 164/211) (Fig. 1).

No meaningful correlation was found between the affected anatomical regions and BMI, physical activity, or weekly working hours.

The analysis reveals gender-specific differences in the frequency of muscular complaints in different anatomic regions. The strongest difference is observed in neck complaints: 96% (*n* = 380/395) of women report issues in this area, compared to 88% (*n* = 313/354) of men. A substantial difference is also in upper back complaints, with 72% (*n* = 278/389) of women affected, versus 58% (*n* = 204/352) of men.

**Fig. 2:**
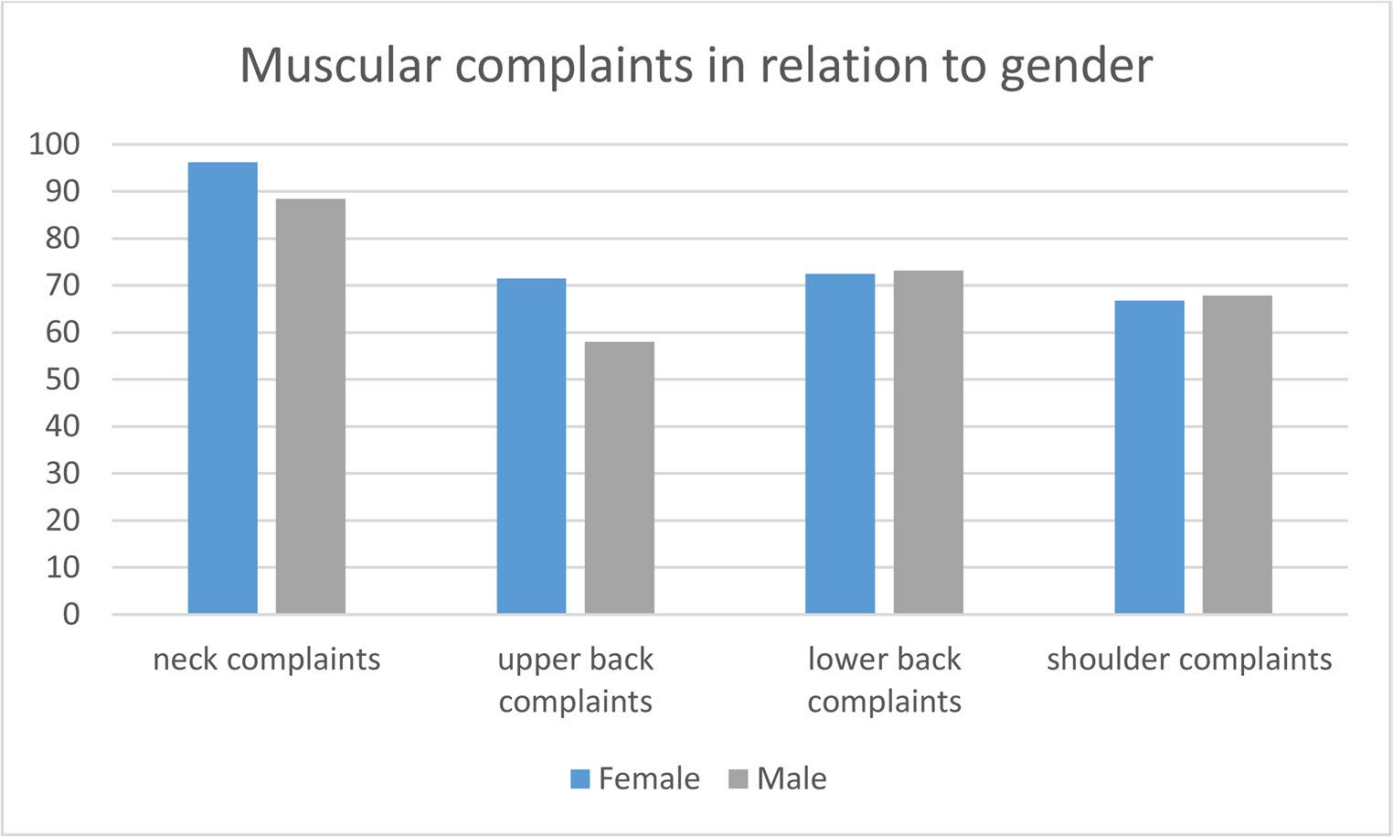
Muscular complaints in percent per group in relation to gender

In contrast, complaints related to the lower back and shoulder show similar results (Fig. 2).

## Discussion

### Participant demographics and work characteristics

This study provides an unfiltered depiction of the burden of musculoskeletal disorders among ENT physicians in Germany. In contrast to many other prevalence studies conducted in different countries, our study includes an exceptionally high number of participants, with a total of 751 respondents [5, 6, 9]. Calculating an exact response rate is nearly impossible, as we do know the total number of ENT specialists practicing in Germany (2023: 6608 [11]), but not how many of them are subscribed to the newsletter of the German Society of Otorhinolaryngology, through which the questionnaire was distributed. The distribution of respondents regarding the outpatient and inpatient sectors resembles the data from the German Medical Association from the year 2023 [11]. Here, 4609 worked in outpatient care, 1617 in inpatient care, and the rest, for example, in authorities. The gender distribution among the participants was very similar, with 53% women and 47% men. The proportion of women is slightly higher than in the data published by the German Medical Association in 2023, which reported about 40% female ENT doctors in practice [11]. The average age of the participants, 51 years, also corresponds to the age distribution of practicing ENT doctors in Germany announced in 2023. Here the largest proportion falls within the 40–59 age group [11]. The average BMI given here, at 24, is slightly below that of the German population according to 2021 data from the Federal Statistical Office, which reports an average BMI of 26 [12]. The average number of working hours per week among the respondents was 40.3 h, which is above the average weekly working time of all employed persons in Germany in 2023, which was 34.3 h [13]. It is possible that longer working hours are associated with increased physical load [14]. On average, the questionnaire participants engaged in just over three hours of sport per week, which was slightly below the average of four hours reported for the German population [15]. *Surprisingly*,* no significant difference between operating and non-operating ENT specialists was found. A possible explanation is that even non-surgical ENT physicians often perform procedures or examinations—such as endoscopy*,* microscopy*,* or working with patients in constrained postures—that require prolonged static or asymmetric positioning. Additionally*,* self-selection bias and the relatively small sample sizes within subgroups may reduce the statistical power to identify significant differences.*

### Prevalence of musculoskeletal complaints among ENT specialists

The most frequently affected anatomical region was the neck, with 93% of respondents reporting lifetime prevalence of complaints and 82% reporting complaints in the past twelve months. *In line with these findings*,* 88% of surveyed ENT physicians in Canada also reported experiencing neck pain* [5]. *Whereas in Ireland*,* only 59% of respondents reported neck pain* [6]. This remarkably high prevalence stands in contrast to that of the general German population, which, according to the RKI’s (Robert-Koch-Institute) Burden 2020 study, reported a twelve-month prevalence of neck pain of 46% [16]. This discrepancy may be attributable to the substantial strain placed on the neck musculature of ENT specialists due to the frequent use of endoscopes and microscopes [17]. This is consistent with the data on upper back complaints, a region that is also subjected to strain during the use of microscopes and endoscopes [18]. In this study, 55% of respondents reported upper back complaints within the past twelve months, whereas data from the general population presented in the RKI’s Burden 2020 study showed a twelve-month prevalence of 27% for upper back complaints [16]. In contrast, the prevalence of lower back complaints among the surveyed ENT specialists was 56%, which is comparable to the twelve-month prevalence of 53% reported in the general population, according to data from the Burden 2020 study by the Robert Koch Institute [16]. This finding supports the assumption that the cervical and upper thoracic musculature in otolaryngologists may be exposed to greater occupational strain than in the general population, likely due to prolonged static postures during the use of microscopes and endoscopes [17, 18]. In contrast, the lumbar region does not appear to be subject to increased occupational load beyond that commonly observed in the broader population. With an increasing number of years in the profession (Fig. 1), the prevalence of complaints also appeared to be higher. This may also be related to the fact that greater professional experience is associated with older age. In summary, the present findings demonstrate a markedly high prevalence of neck and upper back complaints among ENT specialists—considerably higher than in the general population. This raises important questions regarding the consequences of these musculoskeletal issues. *Similar*,* though overall slightly lower prevalence rates have been reported in studies investigating physicians who also work with magnification aids such as loupes or microscopes. One study examining the burden of musculoskeletal disorders among dentists in China reported a prevalence of neck pain in 84% percent of respondents* [19]. *A review of the literature reported that 62% of neurosurgeons experienced neck pain across the studies analyzed* [20]. *Considering non-medical professions*,* a significantly lower prevalence was found among office workers. In one study*,* 46% reported experiencing neck pain within the past 12 months* [21].

### Functional impact and need for preventive measures

In regard to the consequences of these musculoskeletal issues, 56% of respondents reported limitations in everyday activities, and 66% reported restrictions during physical or sporting activities. This is concerning, as physical activity is widely recognized as a protective factor against musculoskeletal disorders [22], indicating a potential negative feedback loop. In line with this a currently ongoing study in our research group provides preliminary evidence supporting this, indicating that targeted training can reduce muscular tension in ENT surgeons during surgery. Moreover, the occupational musculoskeletal burden appears to have consequences not only in private life but also in professional functioning. A total of 53% of participants reported feeling restricted in their work activities due to these complaints, and 22% had already taken sick leave as a result. These findings underscore the urgent need for preventive measures—not only to preserve professional performance and occupational health but also to maintain quality of life and functional capacity in daily life. This is further underscored by the fact that a considerable proportion of respondents are currently undergoing treatment for their complaints: 11% reported the use of analgesic medication, 41% are receiving or received physiotherapy, and 3% have already undergone surgical intervention. As illustrated in Fig. 1, the prevalence of musculoskeletal complaints appears to increase with the number of years in professional practice. This trend emphasizes the importance of implementing early preventive measures to support long-term occupational health and ensure sustained professional longevity. In line with data from the Burden 2020 study, female participants in the present survey reported slightly higher rates of symptoms in the neck and upper back regions compared to their male counterparts [16].

The survey demonstrates the high prevalence of work-related musculoskeletal strain among ENT specialists, as well as the significant impact of these complaints. Naturally, it cannot be ruled out that some degree of bias may exist due to symptoms caused by non-occupational causes. To minimize this risk, participants were explicitly instructed to report only those complaints not associated with sports injuries or similar non-work-related incidents.

Furthermore, comparison with epidemiological data from the general population reveals a clear difference, indicating a substantially higher burden among ENT specialists—most likely attributable to work-related factors. These findings underline the urgent need to raise awareness for these work-related health issues and to implement preventive strategies aimed at reducing musculoskeletal complaints in this professional group.

### Strengths and limitations

In line with the general challenges of a survey, the present study experienced a high rate of non-respondents. A potential bias can be identified here, as those particularly affected might be more likely to respond. This means that ENT doctors experiencing issues may have been more inclined to participate, while those without any problems might not have felt addressed by the questionnaire. To mitigate this, the corresponding cover letter explicitly encouraged for participation even in the absence of any complaints. *An important limitation is the distinction between subjective complaints and clinically verified illnesses*,* as the clinical relevance of reported symptoms remains uncertain and they do not necessarily correspond with objective medical findings.*

## Conclusion

This study provides the first large-scale assessment of work-related musculoskeletal disorders among ENT physicians in Germany, involving 751 respondents. The data show a notably high lifetime prevalence of neck complaints (93%) and substantial rates of shoulder and back pain. Musculoskeletal symptoms increased with professional experience and led to considerable limitations in work and daily activities, with over half reporting impaired work performance and nearly a quarter taking sick leave. Female ENT physicians experienced higher rates of neck and upper back issues than their male counterparts. These findings highlight the critical need for targeted prevention strategies to reduce musculoskeletal strain, improve working conditions, and preserve the health and career sustainability of ENT specialists.
